# The London Hospital

**Published:** 1852-11

**Authors:** 


THE LONDON HOSPITAL. *Physicians*—Drs. Cobb, Little, and Pereira. *Assistant Physicians*—Drs. Fraser, Davies, and Parker. *Consulting Physicians in Obstetric Cases*—Dr. Ramsbotham. *Surgeons*—Messrs. Luke, Adams, and Curling. *Assistant Surgeons*—Messrs. Critchett, N. Ward, and Wordsworth.

MEDICAL AND SURGICAL SCHOOL. *Winter Session.*—Medicine, Dr. Little. Surgery, Messrs. Curling and Critchett. Descriptive and Surgical Anatomy, Mr. J. Adams. General Anatomy and Physiology, Dr. Carpenter. Practical Anatomy, Messrs. N. Ward, and Brown. Chemistry, Mr. Letheby. Anatomy and Pathology of the Teeth and Dental Surgery, Mr. Barrett. *Summer Session.*—Midwifery and Diseases of Women and Children, Dr. Ramsbotham. Forensic Medicine, Drs. Ramsbotham and Letheby. Materia Medica and Therapeutics, Dr. Davies. Practical Chemistry, Dr. Letheby. Botany, Mr. Bentley, Comparative Anatomy, Dr. Carpenter.

